# Whole-genome risk prediction of common diseases in human preimplantation embryos

**DOI:** 10.1038/s41591-022-01735-0

**Published:** 2022-03-21

**Authors:** Akash Kumar, Kate Im, Milena Banjevic, Pauline C. Ng, Tate Tunstall, Geronimo Garcia, Luisa Galhardo, Jiayi Sun, Oren N. Schaedel, Brynn Levy, Donna Hongo, Dusan Kijacic, Michelle Kiehl, Nam D. Tran, Peter C. Klatsky, Matthew Rabinowitz

**Affiliations:** 1 MyOme, Inc., Menlo Park, CA USA; 2 Natera, Inc., San Carlos, CA USA; 3 Department of Pathology and Cell Biology, Columbia University Irving Medical Center, New York, NY USA; 4 Spring Fertility, San Francisco, CA USA

**Keywords:** Predictive medicine, Diagnostic markers, Genomics

## Abstract

Preimplantation genetic testing (PGT) of in-vitro-fertilized embryos has been proposed as a method to reduce transmission of common disease; however, more comprehensive embryo genetic assessment, combining the effects of common variants and rare variants, remains unavailable. Here, we used a combination of molecular and statistical techniques to reliably infer inherited genome sequence in 110 embryos and model susceptibility across 12 common conditions. We observed a genotype accuracy of 99.0–99.4% at sites relevant to polygenic risk scoring in cases from day-5 embryo biopsies and 97.2–99.1% in cases from day-3 embryo biopsies. Combining rare variants with polygenic risk score (PRS) magnifies predicted differences across sibling embryos. For example, in a couple with a pathogenic *BRCA1* variant, we predicted a 15-fold difference in odds ratio (OR) across siblings when combining versus a 4.5-fold or 3-fold difference with *BRCA1* or PRS alone. Our findings may inform the discussion of utility and implementation of genome-based PGT in clinical practice.

## Abstract (web summary)

A computational approach combining whole-genome sequencing of parental genomes and genotyping of preimplantation embryos allows accurate prediction of the inherited genomes of embryos and calculation of polygenic risk scores.

## Main

**Fig. 1:**
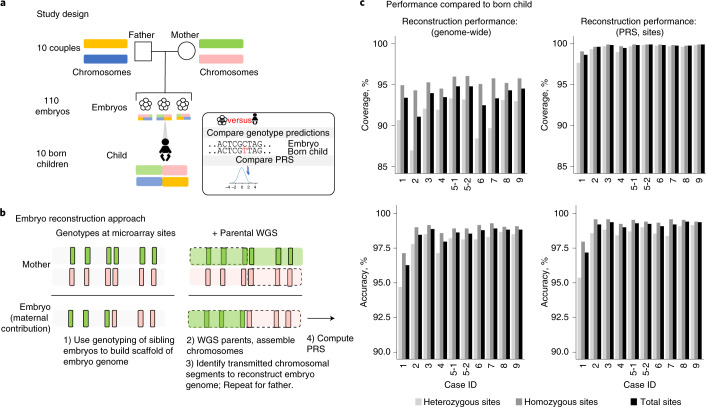
WGR and approach. **a**, This research study involved reconstruction of 110 embryo genomes from 10 couples and comparison to the genome sequence of the born child. Twelve PRS models were computed from the born-child samples and the 10 corresponding reconstructed embryos and compared for concordance. **b**, WGR involves whole-genome sequencing (WGS) of prospective parents and single-nucleotide polymorphism (SNP) microarray genotyping of sibling embryos (Methods and Supplemental Note 1). Allele measurements at each SNP are color-coded based on the parental haplotype of origin illustrated in **a**. A combination of molecular and statistical/population-based techniques phase the parents’ chromosomes, infer the locations of meiotic recombination for each embryo and correct errors introduced in the process of testing single-cell or few-cell embryo biopsies (Methods). Reconstructed embryo whole genomes are used to predict common disease risk by calculating PRSs and inferring the inheritance of rare variants with high impact on disease risk. **c**, Performance by comparing genotypes from WGR with the born child’s DNA shows genotype accuracies ranging from 99.0% to 99.4% at sites used in polygenic prediction in day-5 embryos and 97.2% to 99.1% in day-3 embryos. Case 1 includes only day-3 embryos, and case 2 includes both day-3 and day-5 embryos. All other cases included day-5 embryos only. Statistics are subdivided by genotype (heterozygous or homozygous) in the born child.

PGT enables profiling of embryos for family-specific genetic disorders before implantation. Although PGT is currently used in preventing rare mendelian disorders^1,2^, several groups have explored expanding testing to include common conditions, such as heart disease and cancers^3–6^. These approaches rely on the use of a PRS^7^ that combines the effects of tens or hundreds or thousands of genetic variants into a single predictor. However, due to the limited quantity and quality of DNA in single-cell or few-cell embryo biopsies, attempts to comprehensively profile the genomes of embryos are costly and time intensive^8–10^, suffer from inaccuracies related to allele dropout, require extended relatives^2^ or rely on imputation, which hampers the detection of rare deleterious variants in genes like *BRCA1*^11^. To overcome these limitations, we have extended a strategy^1^ for whole-genome reconstruction (WGR) that uses parental genome sequencing and embryo genotyping to predict the inherited genome sequence of an embryo. Here, we apply this approach to 110 embryos across 10 couples and compute polygenic predictors across 12 medical conditions, including cancers, cardiometabolic and autoimmune diseases (Fig. 1). The reconstructed genome and polygenic predictions for the embryos were compared to those generated from a tissue sample of the corresponding born children.

## Results

**Extended Data Fig. 1:**
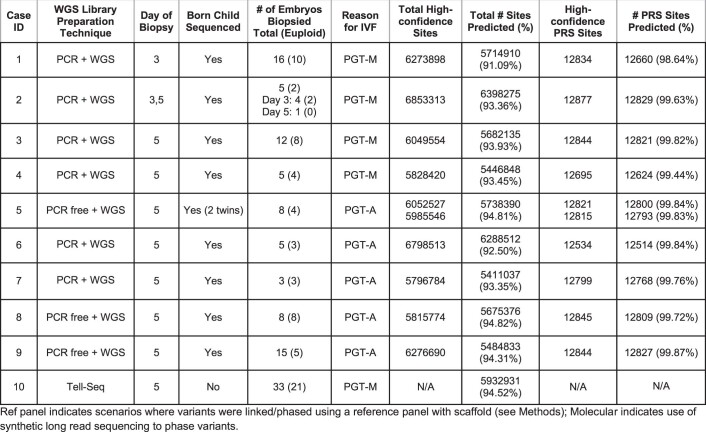
Summary of PGT assays performed. A total of 29 individuals were sequenced on a variety of sequencing platforms. PGT from embryo biopsies were performed by a commercial lab (Natera, formerly Gene Security Network) on the HumanCytoSNP-12 BeadChip array, ranging from 3 to 33 embryos. Coverage and accuracy assessed at genomic positions that are high-confidence genotype calls in parents and born child.

**Extended Data Fig. 2:**
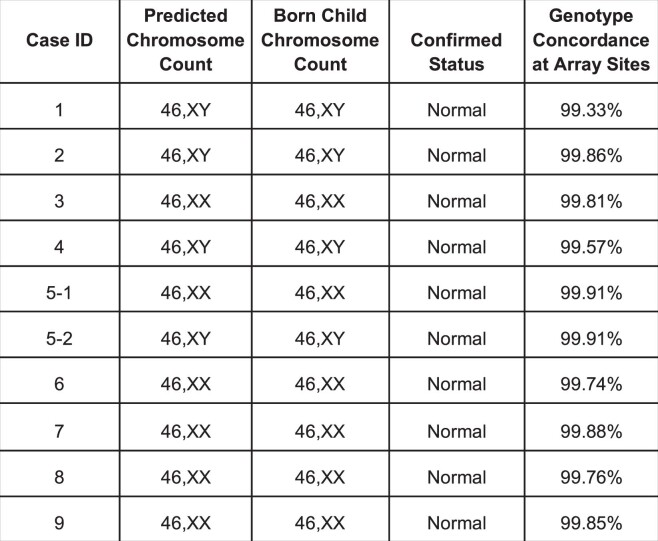
Prediction of aneuploidy and genotypes across embryos and comparison with born children. Genotype predictions at chromosomal microarray positions for each embryo were compared to genotypes measured from WGS of the born child. Predictions obtained using SNP Array and Parental Support.

Samples used in this study were obtained from couples that previously underwent in vitro fertilization (IVF) with prior clinical PGT (Extended Data Fig. 1) under an institutional review board (IRB) protocol (Methods). DNA was available from 10 born children who had prior PGT results available. Clinical PGT for aneuploidy (PGT-A) revealed 68 of the 110 embryos were euploid, and 42 of 110 embryos had one or more aneuploid chromosomes (Extended Data Fig. 1 and Extended Data Fig. 2). We achieved WGR of embryos by performing high-coverage genome sequencing of both parents and array measurements of sibling embryos (Methods and Supplemental Note 1). We used a combination of molecular and statistical approaches to link parental variants into ‘haplotypes’ corresponding to individual euploid chromosomes, determine sites of meiotic recombination for each embryo and assemble the relevant haplotype segments between each of the recombination sites to approximate the entire inherited embryonic genome^1^.

**Extended Data Fig. 3:**
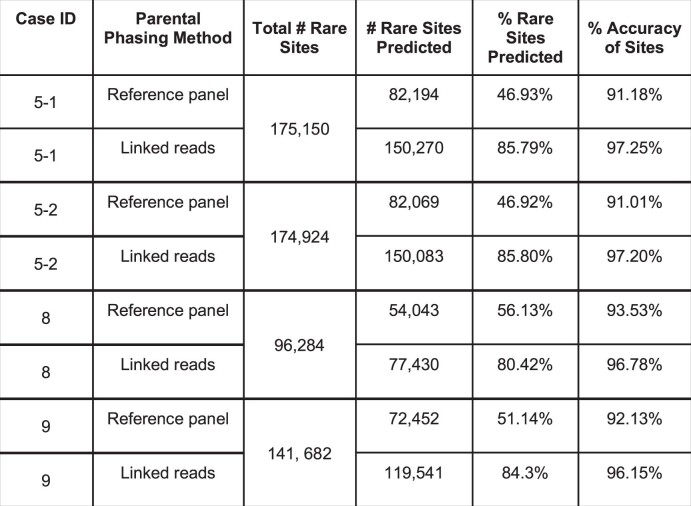
Performance of whole-genome reconstruction on individually rare variants using synthetic long-read sequencing as a method to phase parents. This method was performed on the parental genomes in 4 cases (Case ID 5, 8, 9 and 10). Accuracy could not be assessed for Case ID10 as no born child was available for comparison. Variants with an allele frequency <0.1% or not present in the gnomAD database were considered rare. Only high-confidence variants (as described in Methods) from both Tell-Seq and PCR-free WGS protocols were evaluated.

We evaluated the accuracy of WGR by comparing our genotype predictions to the actual genotypes present in the born children. We predicted an average of 5.8 million sites in embryos, ranging from 5.4 to 6.4 million variants (Fig. 1, Extended Data Figs. 1 and 2 and Supplemental Table 1). Genome-wide prediction accuracy ranged from 96.3–98.4% in day-3 embryo biopsies to 98.0–98.9% in day-5 embryo biopsies compared to the born child. We posit that processing and testing of single cells from day-3 blastomere biopsies yields diminished performance compared to processing and testing of multiple cells from day-5 trophectoderm biopsies. In four couples, we also performed a modified form of library preparation using long fragments of DNA (synthetic long-read sequencing (Transposase Enzyme Linked Long-read Sequencing (TELL-Seq)); Extended Data Fig. 1 and Extended Data Fig. 3) to capture the phase of rare variants (defined as allele frequency less than 0.1%) and increased the number and accuracy of rare variants predicted in each family (Extended Data Fig. 3).

**Fig. 2:**
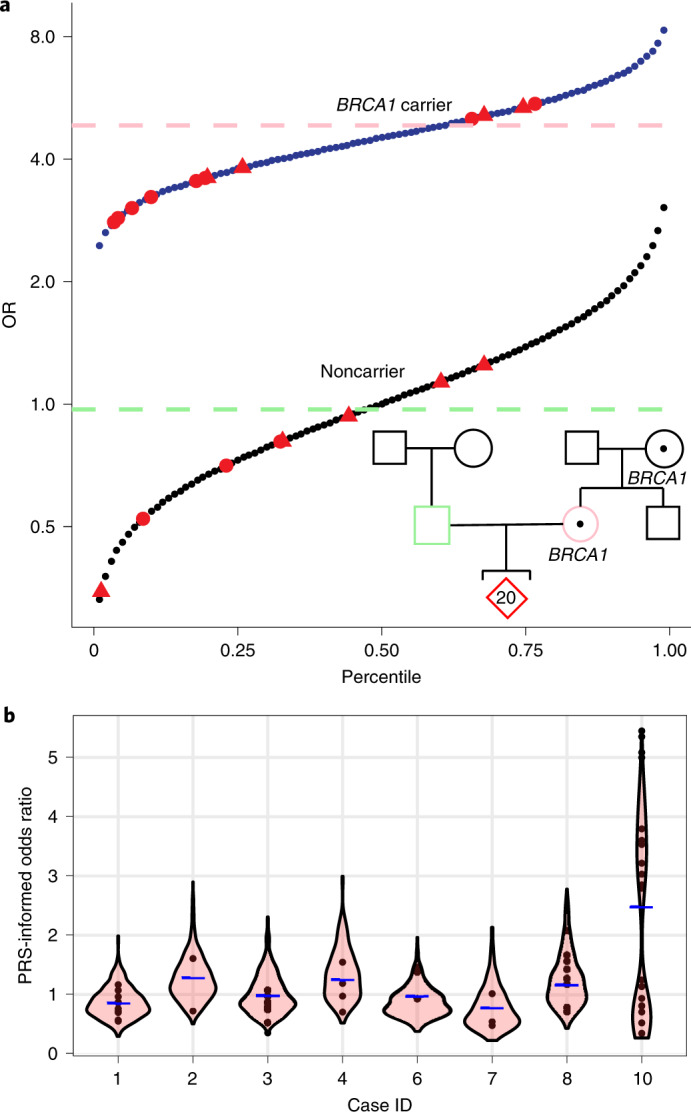
Intra- and interfamilial differences in predicted genetic risk for breast cancer susceptibility. **a**, Integrated polygenic/monogenic prediction in research participants with family history of breast cancer, *BRCA1* variant and multiple embryos (red). Thirteen embryos carried a pathogenic *BRCA1* variant. Using a logistic model fit on over 22,000 individuals in the UKB with relevant clinical and genetic information using PRS and carrier status as separate variables, we predicted the odds of disease for each embryo as per Fahed et al.^12^. The blue line indicates OR as a function of PRS for *BRCA1* carriers, and the black line indicates OR for noncarriers. The method accounts for the reduction in effect of PRS in the context of positive *BRCA1* status, captured in the difference in slope of the two lines. The female participant’s PRS is shown as a pink dashed line, and the male participant’s PRS is shown as a green dashed line (projected as female risk for comparison). Similarly, male embryos (triangles) are shown separately. **b**, Genomic risk for breast cancer is shown across ten couples and their embryos. Predicted genetic disease risk for each embryo (circles) is shown along with a violin-plot distribution of results from 500 simulated embryos (as described in Methods), with average parental PRS shown as a blue dash. Inheritance of a pathogenic *BRCA1* variant (**a**) accounts for increased variability and a bimodal distribution in breast cancer disease risk for case 10.

**Extended Data Fig. 4:**
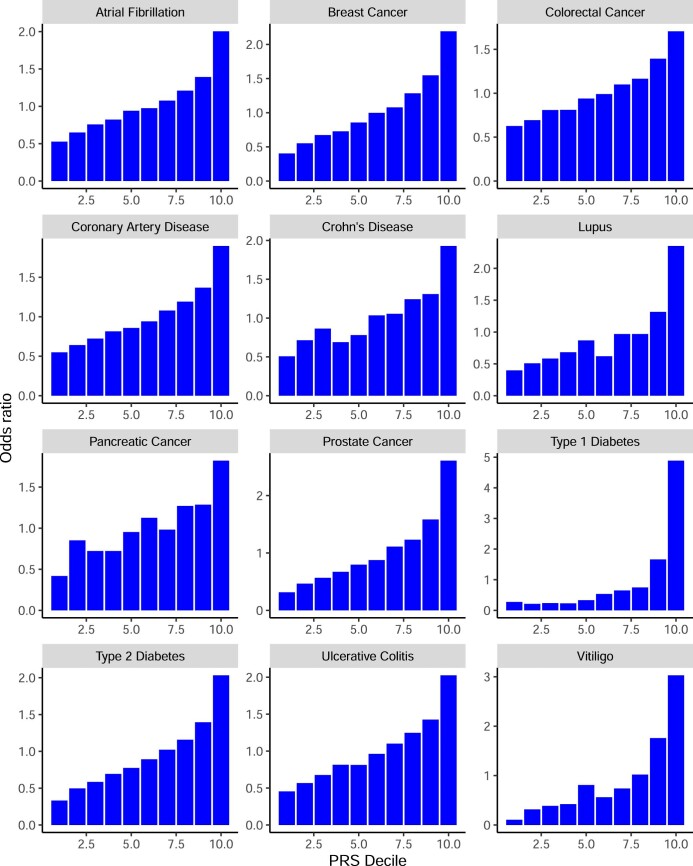
Performance of polygenic risk score in UK Biobank cohort of adults. Empirical odds ratios per decile of PRS score calculated in British White individuals in the UK Biobank.

**Extended Data Fig. 5:**
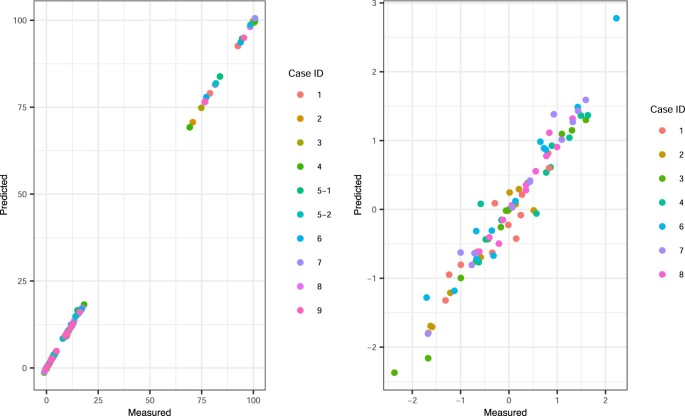
Correlation of polygenic risk score from embryo predictions and born child. **a**, Illustrates the close correlation between predicted and measured (born child) raw polygenic risk score, consistent with genotype concordance between predicted and measured polygenic risk. **b**, Correlation between predicted and measured z-score derived from raw polygenic risk score (r^2^=0.947). Case IDs 5 and 9 were excluded from this analysis as the approach to mean-center polygenic risk using population ancestry is unable to account for admixture.

**Extended Data Fig. 6:**
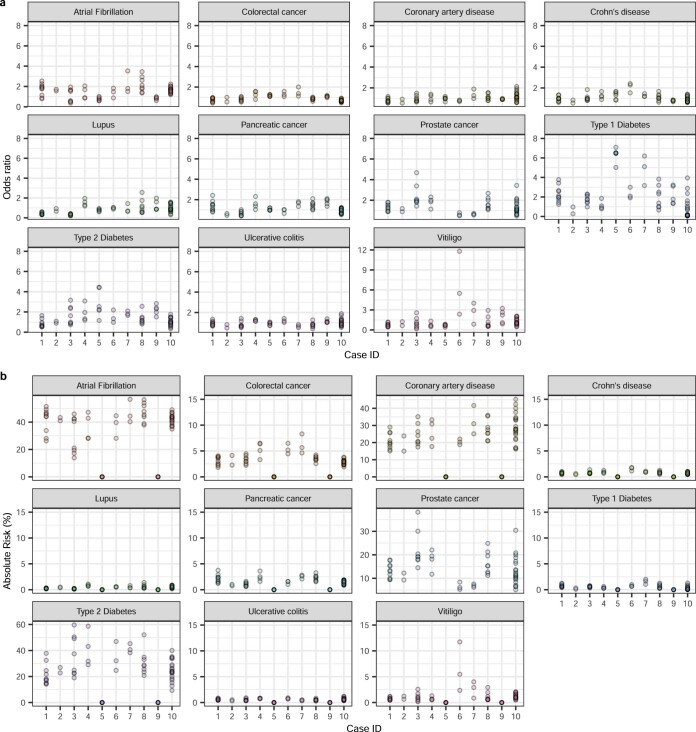
Variability in predicted disease risk across 11 additional conditions. **a**, Predicted relative risk of disease. **b**, Predicted absolute disease risk. Autoimmune conditions (Type 1 diabetes, Vitiligo) exhibit greater variability in predicted disease risk as has been previously reported. Note: Risk for the embryo in case ID 7 carrying the *APC* risk allele, rs1801155, is based solely on that allele. A competing hazards method (Gail et al 1989) was used to calculate the absolute risk for atrial fibrillation, coronary artery disease, prostate cancer and Type 2 diabetes. Age- and ethnicity-specific overall and disease specific mortality rates were taken from the CDC Wonder database (Underlying Cause of Death 1999–2019). Individuals with >20% admixture of African/Asian ancestry as determined using ELAI software were not considered in this analysis.

Our approach enabled the prediction of both rare and common variants in embryo genomes. To explore the impact of combining these variants when predicting common disease risk, we used the reconstructed embryo genomes to calculate PRSs. For each embryo, we calculated the risk scores for a set of published polygenic models that we validated and calibrated in the UK Biobank (UKB) on a population-specific basis (Methods, Extended Data Fig. 4 and Supplemental Table 2). Using high-confidence positions called from WGS of born children as ‘truth’, we observed a genotype accuracy of 99.0–99.4% at sites relevant to polygenic risk scoring in cases from day-5 trophectoderm biopsies and 97.2–99.1% in cycles containing day-3 blastomere biopsies (Extended Data Fig. 1 and Fig. 1). We normalized the PRSs of each embryo to account for population structure and converted scores into predicted odds of disease using a logistic regression model (Methods). Correlation between the residualized PRS computed from embryo biopsies and those computed from born-child samples was *r*^2^ = 0.947 (Extended Data Fig. 5). We observed variability in polygenic disease risk across embryos of different families and among the sibling embryos within families (Fig. 2 and Extended Data Fig. 6). The most variation in predicted OR was observed in autoimmune disorders, presumably due to the larger fraction of variants with high impact in the PRS models. Whereas vitiligo and type 1 diabetes displayed the highest ORs, the difference in absolute risk across sibling embryos in these cases was less than 10% due to the relative rarity of the diseases. The largest difference in absolute risk was seen in more common cardiometabolic diseases.

We next examined the potential impact of using monogenic and polygenic variants in the context of PGT. In one couple with a family history of breast cancer undergoing PGT for monogenic diseases, we confirmed a previously identified pathogenic *BRCA1* variant in the mother. Of the family’s 20 euploid embryos, we predicted 13 embryos carried the pathogenic *BRCA1* variant. After genome reconstruction, we combined the effect of carrying a pathogenic *BRCA1* or *BRCA2* variant with that of a polygenic model using a logistic regression as described in Fahed et al.^12^ to calculate a single risk prediction for each embryo and account for change in the PRS effect size in the context of positive *BRCA1* status. We calculated the PRS OR per standard deviation was 1.3 among carriers and 1.6 among noncarriers. Predicted genetic risk for breast cancer varied by 15-fold across the embryos, with OR ranging from 0.35 (non-*BRCA1* carrier with low PRS) to 5.35 (*BRCA1* carrier with high PRS) (Fig. 2a). We explored whether use of PRS alone, as currently available in PGT for polygenic disorders, may lead to inadvertent transfer of a high-risk embryo. In this case, of 14 embryos with PRS below the 50th percentile, 9 carried the pathogenic *BRCA1* variant. We next compared predicted breast cancer risk estimates across embryos derived from the remaining couples. Aside from those embryos with *BRCA1* mutations, most embryos were predicted to have less than twofold OR of breast cancer (Fig. 2b), suggesting that PGT for breast cancer is most beneficial in scenarios with a pathogenic variant with high impact on disease risk^5,6^. One couple not undergoing PGT for monogenic diseases was incidentally found to carry an *APC* risk allele (rs1801155) associated with a twofold increased risk of colorectal cancer. After genome reconstruction of this couple’s three euploid embryos, one embryo was predicted to harbor the risk allele in *APC* (Extended Data Fig. 6). Both of the rare variants that we identified in these well-established colon (*APC*) and breast cancer (*BRCA1*) genes would likely be missed by imputation, subject to the reference population used. Blind to knowledge of a pathogenic variant, a couple with a family history of disease may unintentionally prioritize a mutation-positive embryo for implantation based on PRS alone. The frequency of this scenario depends on the condition and family history. For example, breast cancer inherited genetic mutations are estimated to account for between 5% and 10% of cases, with higher estimates in certain populations^13^ and those with family history.

## Discussion

In this preclinical research study, we demonstrated that WGR for embryos is feasible and enables accurate calculation of PRS when compared with results from genomic samples from 10 born children. We observed heterogeneity in predicted disease risk across families and across siblings. PRS on many common variants with small effect sizes, in combination with rare variants may better capture the heterogeneity in predicted disease risk.

Several areas for improvement remain. First, our approach is limited to inherited genetic variation. Although we confirmed inheritance at several million sites in a born child, we excluded variants that may be ‘new’ or de novo in the gametes or somatic mutations that arise after conception. Although rare, de novo variants represent a substantial portion of certain early-onset neurodevelopmental disorders, including autism and intellectual disability. Our predictions were more accurate in day-5 embryos compared with day-3 embryos, likely due to increased cellular material present in a trophectoderm biopsy. As WGS costs decrease, we anticipate our approach can be supplemented with ultradeep sequencing of embryo biopsies to detect these variants.

Second, PRSs have limited effectiveness in non-European populations. Human genetics research historically involved participants of European ancestries, and as a result, the predictive power in non-European cohorts is limited. In the long term, strategies including recruitment of ancestrally diverse cohorts and fine-mapping to the causative variants should improve performance across ancestries. In the short term, using a subset of models with evidence of cross-ancestry performance may be necessary.

Third, the clinical utility of using PRS to reduce disease risk in preimplantation embryos remains to be proven. Findings from research cohorts may not generalize to sibling embryos in IVF and reduce our prediction accuracy. These biobanks are predominantly composed of unrelated individuals; several groups have reported a decrease in the predictive ability of models trained using unrelated individuals within families particularly for cognitive and psychiatric traits^14^. Our analysis of a cohort of siblings versus unrelated individuals in the UKB, used to generate the respective OR among sibling embryos (Supplemental Note 3), resulted in similar breast cancer PRS effect sizes for siblings versus unrelated individuals. More family-based prospective studies of genetics and disease are needed to alleviate these concerns. Furthermore, independent of the intrafamily effect, children born at present may face a different environment and risk profile than adult participants in most research biobanks. Separately, individuals with family history of disease may exhibit a higher absolute risk reduction (and lower relative risk reduction) of disease due to higher a priori risk of disease^6,15^.

Fourth, incorporating polygenic information into prenatal decision-making raises ethical and practical questions that deserve consideration in an already complex field^15,16^. Foremost is the risk of unequal access to this technology for families due to either cost of IVF or limitations in cross-ancestry performance of polygenic models. Our studies are free to participants and involve several models validated across multiple ancestries. However, widespread disparities must continue to be addressed. Separately, Turley et al. highlighted the complexity of communicating expected risk (both relative and absolute) as well as the uncertainty involved in these estimates^15^. We agree with Turley et al. on the need for clear and accurate pretest communication; those study participants who received embryo predictions first completed a pretest counseling and consent session with a medical geneticist and then completed a follow-up survey to gauge their understanding and perceived benefit. Despite limitations, emerging evidence suggests public interest in incorporating PRS in embryo screening; a recent survey found 68% of 1,457 US participants believed embryo screening using PRS was reasonable^17^. Further study, through research protocols like ours, can help generate much-needed evidence on utility.

Fifth, a subset of individuals in this study were referred for aneuploidy screening using PGT-A, an adjunct intervention in IVF^18^. The impact of chromosomal mosaicism from a blastocyst biopsy on the clinical effectiveness of PGT-A remains under debate^18–20^. Additionally, our approach does not address the accuracy of PRS testing in embryos found to be mosaic or aneuploid during PGT-A.

As our understanding and the predictive power of PRSs improves and sequencing technology becomes more cost effective, this approach may be used to reliably infer inherited genome sequence and model predicted genetic risk in embryos of couples with a personal and/or family history of common disease undergoing IVF.

## Methods

### Recruitment of participants, sample collection and sequencing

All participants were recruited through one of several IRB studies (E&I West Coast Board IRB protocol 10176) and WCG IRB protocols 20180294 and 20202676. Appropriate consent was obtained from each participant, and a subset of individuals participating in protocol 20180294 consented for return of results to receive certain incidental single-gene findings on themselves and a report on euploid embryos. For some of the couples whose data were included in the analyses, we had prior knowledge on the presence of rare pathogenic variants. For each individual, we extracted genomic DNA from either whole blood or saliva samples, as available. We fragmented DNA, prepared and sequenced shotgun-sequencing libraries using Illumina or BGI kits following manufacturer’s instructions in either paired-end 100-bp (case 4) or 150-bp read configuration. We targeted an average depth of 30× for WGS on parents’ and born children’s DNA. Actual mean coverage for all samples was ≥29 and ranged from 29× to 111× (Supplemental Table 3). Individuals participating in protocol 20180294 (prospective study) also had the option to receive certain incidental single-gene findings on themselves and a report on euploid embryos that included polygenic disease risk information at no additional cost to their IVF cycle. Individuals who elected to receive polygenic risk information received both pretest and posttest counseling with a medical geneticist or genetic counselor which included a discussion of how polygenic models are constructed, the experimental nature of predictions made and visualizations of polygenic risk.

In four families with fresh blood samples available, we additionally performed synthetic long-read sequencing on both individuals in the couple. Modifications to our protocol included high-molecular-weight DNA extraction (Circulomics) and library preparation using a TELL-Seq library with standard protocols, except for reduced transposable enzyme.

### WGS, alignment and genotyping

WGS primary and secondary analyses were performed according to the Broad Institute’s best practices pipeline (GATK), implemented by Sentieon Software (Sentieon). Briefly, we mapped reads to the human reference genome sequence (GRCh37) with bwa v0.7.17. Genotyping involved two steps. First, we performed joint variant calling on the parents and the born child using Sentieon’s GVCFtyper and filtered these based on internal quality-control thresholds, including base quality ≥20, read depth ≥8, Fisher strand bias <30 and quality by depth >4. Second, we called genotypes at sites specific to polygenic models with a read depth of at least 8×. Sequence reads from case 4 were also reported in our previous publication^1^.

### Embryo genotyping and PS analysis

To genotype embryo biopsy specimens, we extracted and amplified DNA, followed by genotyping using a rapid SNP microarray protocol on Illumina’s HumanCytoSNP-12 BeadChip. We combined sibling embryos’ and parents’ SNP microarray measurements in a two-step method termed parental support (PS; Supplemental Note 1). First, we used a statistical model to determine the maximum likelihood estimate phase of heterozygous single-nucleotide variants in each parent by combining recombination frequencies from the HapMap database with SNP array measurements from parents and SNP array measurements from sibling embryos. This will be referred to as PS haplotypes. Second, we determined PS embryo genotypes using a hidden Markov model (HMM) that finds the most likely parental haplotype transmitted to each embryo given SNP array measurements from the embryo and maximum likelihood estimate phase for each parent (Supplemental Note 1). The outputs of the HMM informed the meiotic recombination sites. As these samples were processed on the same workflow used in a clinical PGT-A platform (Natera), we relied on the established Natera Spectrum pipeline to identify embryos with aneuploid chromosomes^20^. This platform can detect copy-number variants down to 5 Mb.

### Haplotype phasing of parents

**Extended Data Fig. 7:**
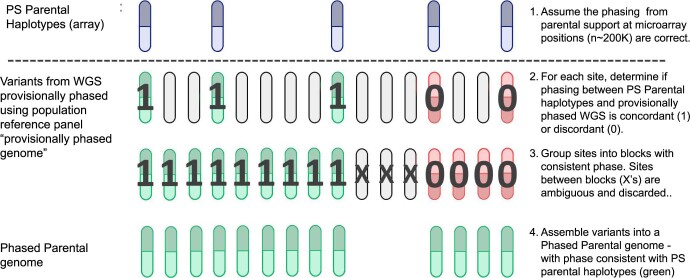
Obtaining a phased parental genome. Each parent’s genome is phased using PS parental haplotypes (see Supplemental Note 1) and population reference panels using SHAPEIT4. The PS parental haplotypes serve as a scaffold (step 1) consisting of approximately 200,000 variants. WGS of both parents are provisionally phased using population reference panels and compared with PS Parental Haplotypes. Overlapping positions between the parental support haplotypes and provisionally phased WGS are marked as having concordant (1) or discordant (0) phase (step 2) and grouped into blocks (step 3). Interval regions between these blocks are suggestive of either meiotic recombination or error in phasing one or both parents; these sites are discarded (positions marked with ‘X’ in step 3). All remaining sites are used in subsequent assembly of the ‘Phased Parental Genome’ (step 4).

To phase WGS-derived variants in each parent, we used SHAPEIT4 (ref. ^11^) with default parameters using the UK10K Imputation Cohort + 1000 Genomes phase 3 (EGAD00001000776) as a reference panel and PS haplotypes as a scaffold (Supplemental Note 1). This scaffold, consisting of ~200,000 phased variants, served to anchor phasing performed using the reference panel (Extended Data Fig. 7). Each chromosome was processed independently and in parallel; all chromosomes were combined thereafter. Multiallelic sites were excluded. To gain additional performance for rare variants not represented by reference panels, we used linked read sequencing of high-molecular-weight DNA (Supplemental Note 2).

### Reconstruction approach

**Extended Data Fig. 8:**
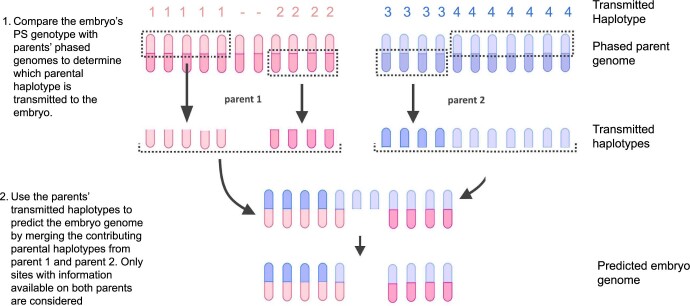
Embryo reconstruction approach. Phased parent genomes and parental support (PS) embryo genotypes are used to reconstruct the embryo’s genome at euploid chromosomes. The phased parent genomes are determined in Extended Data Fig. 7. One parent’s (parent 1) transmitted haplotypes are shown in pink (labeled 1 and 2) and the other parent’s (parent 2) phased haplotypes are shown in purple (labeled 3 and 4). In step 1, the embryo’s PS genotype determines which parental haplotype is transmitted to the embryo (dotted line). Missing data in the embryo or the parents’ phased genomes is excluded. In step 2, positions where genotypes are available from both parents are combined to create the predicted embryo genome; positions with missing data are excluded.

To predict the whole-genome sequence of each embryo, we combined PS embryo genotypes (see above) with phased parental genomes^1^ with the addition of chromosome-spanning haplotypes using an HMM discussed in Supplemental Note 1. The parents’ transmitted haplotype to the embryo was obtained by comparing the PS haplotype ('Haplotype phasing of parents') and the embryo’s PS genotypes ('Embryo genotyping and PS analysis'). We repeated this process across each maternal and paternal chromosome (Extended Data Figs. 7 and 8) with the exception of those chromosomes predicted to be aneuploid using a clinical PGT-A assay (Natera Spectrum).

We filtered low-quality sites in parental and born-child genomes (see above) and multiallelic sites and sites corresponding to a Mendelian error in the sequence data from each family to form a set of ‘high-confidence sites’ that were used to assess coverage and accuracy. We compared predicted embryo genotype calls (derived from reconstruction) with variants called by sequencing of the born child’s DNA.

We annotated high-confidence sites with population allele frequencies from the gnomAD v2.1 dataset, which is composed of approximately 15,000 whole genomes and 125,000 exomes derived from seven populations (African, Latino, Ashkenazi Jewish, East Asian, European, South Asian and other). Variants with an allele frequency <0.1% or not present in the gnomAD database were considered rare.

### Polygenic risk models

#### UKB population

To validate each model independently from the original publication of the PRS, we used genetic and disease information from the ~500,000 individuals in the UKB cohort. For model validation purposes, we separated participants into four groups by self-reported ancestry (field code 21,000; White, Black, East Asian (Chinese) or South Asian) and computed model performance and PRS effect size separately for each group.

#### Phenotype definitions

We used a combination of ICD-9 and ICD-10 codes, self-reported diseases and procedure codes to define each phenotype of interest. A detailed description for all phenotypes is in Supplemental Table 2.

#### Polygenic models

We prioritized previously published polygenic models for each condition of interest which have been tested on at least 1,000 individuals from a broad population (Supplemental Table 4). To validate each model independently from the original publication, we used genetic and disease information from the ~500,000 individuals in the UKB cohort. Variants in the published models that did not have genotype data in the UKB were excluded. Effect sizes (log-transformed ORs) for the remaining variants were taken from the original publication (summarized in Supplemental Table 4). PRSs were calculated as a weighted sum of disease-associated genotypes.

#### PRS effect size

We computed PRS for each individual in the UKB and standardized the score as discussed below. We computed an OR per standard deviation of the risk score using a logistic regression that includes the normalized PRS, age and sex (for breast cancer, prostate cancer and coronary artery disease). We used a variety of metrics, including area under the curve and ORs per decile to evaluate the performance of each model. Models that passed internal quality of area under the curve ≥0.6, increase in OR per PRS decile and an OR ≥ 2 between top and bottom PRS deciles were used in this study. Model performance across deciles can be seen in Extended Data Fig. 3.

#### Centering and standardization

**Extended Data Fig. 9:**
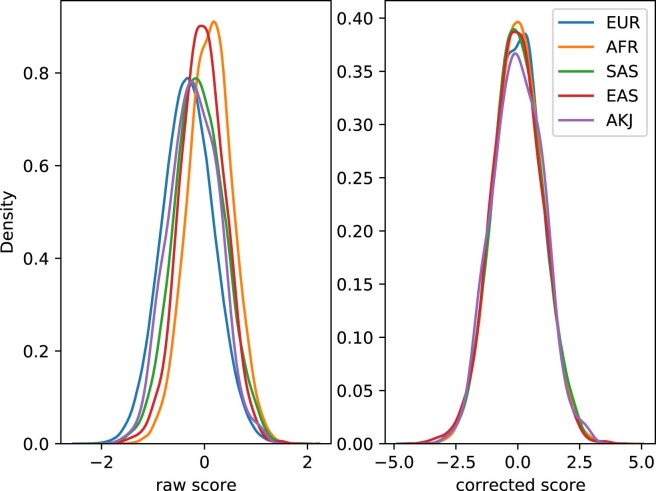
Normalization of polygenic risk scores. Polygenic risk scores before (left) and after (right) centering and standardization for 5 populations within the UK Biobank. Standardized PRS scores have approximately zero mean and unit variance in each population.

To standardize and center PRS to a distribution with approximately zero mean and unit variance, we modified the approach described in Khera et al.^21^. First, we computed principal components (PCs) for individuals in the UKB by projecting their genotypes onto PCs calculated on individuals in the 1000 Genomes Project. We next centered the PRS by subtracting out the PRS value predicted from a linear regression of PRS against the first four PC scores in control individuals (i.e., individuals without the phenotype of interest). This centered PRS was then divided by the standard deviation of the 1000 Genomes Project population most closely related to each individual. Normalized and centered PRSs across multiple ancestries can be seen in Extended Data Fig. 9. PRS scoring in individuals with Ashkenazi Jewish ancestry involved a modified centering process discussed in Supplemental Note 4.

#### Calculating scores in the embryos

We calculated PRSs and ancestral PCs using a similar approach for each reconstructed embryo. In scenarios where we were unable to make a prediction in the embryo, we used the population allele frequency to adjust the score. The score was centered and standardized as described above and transformed into an OR of disease given the PRS. Specifically, OR_PRS_ = *e*^β*PRS^, where *β* is the PRS effect size (i.e., log odds per standard deviation) derived from the UKB (analysis described above) and PRS is the centered and standardized PRS.

#### Integration of monogenic and polygenic risk

We investigated a subset of consenting participants for variants interpreted as pathogenic or likely pathogenic in a predefined set of genes, which included the 59 genes designated by the American College of Medical Genetics as reportable as secondary findings as part of the study. Variants were classified based on American College of Medical Genetics criteria and included manual review of variants annotated in ClinVar as likely pathogenic or pathogenic. Pathogenic/likely pathogenic variants associated with a phenotype for which we report a PRS were incorporated into the risk estimation. For this study, this included *BRCA1* for breast cancer.

After calculating PRS scores in the UKB among women with exome data available, we ran a logistic model fit on female carriers of pathogenic variants in *BRCA1* and *BRCA2* and separately on noncarriers across approximately 22,000 individuals in the UKB. *BRCA1* and *BRCA2* variants were defined from Fahed et al.^12^. The PRS OR per standard deviation was 1.3 among carriers and 1.6 among noncarriers. This information was projected across 99th percentiles with embryos mapped to each category.

### Simulated distribution of PRSs

**Extended Data Fig. 10:**
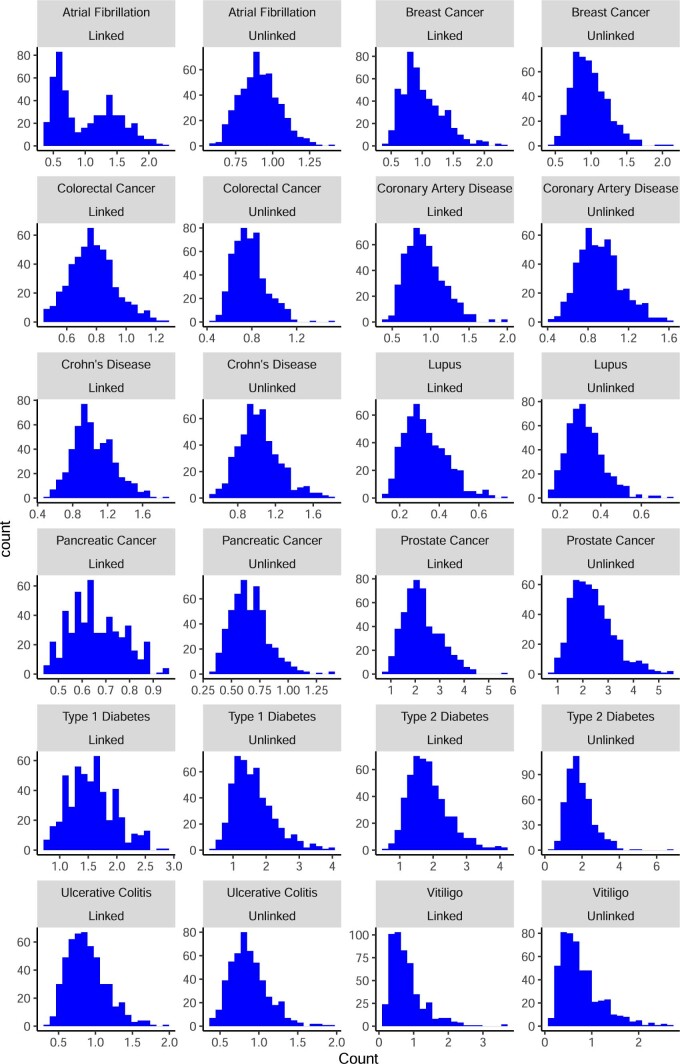
Distributions of simulated embryo risk. Comparison of simulated distributions of odds ratios assuming linked and unlinked neighboring SNPs for one study couple (case ID 3). Atrial fibrillation demonstrates a bimodal distribution with linked SNPs with several simulated children predicted to have an OR of 2, consistent with predictions made on embryos (Extended Data Fig. 6). Simulations on linked SNPs used an approach described in Methods and Caballero et al.^22^.

We simulated embryos (linkage approach) by starting with phased genomes of both parents, adding recombinations between the two mother or two father chromosomes (to approximate meiotic recombination in gametes) and combining these ‘virtual gametes’ at random. We combined PS haplotypes with WGS as discussed above to derive phased parental genomes. We used ped-sim (https://github.com/williamslab/ped-sim) with a pedigree (two parents and one child) and a genetic map (https://github.com/cbherer/Bherer_etal_SexualDimorphismRecombination) to simulate sites for recombination. We intersected the breakpoints derived from ped-sim with the phased parental genomes to generate virtual gametes, combined virtual gametes from the mother and father to generate an embryo genome and calculated polygenic risk in these embryos as discussed above. To generate a distribution of risk scores, we repeat this process 500 times for each couple. In another approach (unlinked approach), we simulated an embryo by choosing one allele from each parent at random and made no assumptions on whether neighboring variants were linked (Extended Data Fig. 10).

### Reporting Summary

Further information on research design is available in the Nature Research Reporting Summary linked to this article.

## Online content

Any methods, additional references, Nature Research reporting summaries, source data, extended data, supplementary information, acknowledgements, peer review information; details of author contributions and competing interests; and statements of data and code availability are available at 10.1038/s41591-022-01735-0.

## Supplementary information

Supplementary Information Supplementary Tables 1 and 2 and Notes 1–4.

Reporting Summary

## Data Availability

Access to primary sequence data from participants of the prospective trial is controlled and cannot be made publicly available. Sequence data from 12 individuals corresponding to four consented couples can be found in EGAS00001005619 and EGAS00001001020. Inquiries on how to access the UK10K imputation reference panel data can be made through the Wellcome Trust Sanger Institute (datasharing@sanger.ac.uk). Information on obtaining approval for access to UKB data is available at www.ukbiobank.ac.uk/researchers. gnomAD data were downloaded from https://gnomad.broadinstitute.org/downloads (s3://gnomad-public-us-east-1/release/).

## Code availability

For whole-genome sequence processing, Sentieon software (including bwa 0.7.17) was used to align sequence reads to genome reference and perform variant calling (https://www.sentieon.com/). bcftools v.1.11–34 was used for variant filtering. To calculate PRSs, plink v1.9 and v2.0 (https://zzz.bwh.harvard.edu/plink/) were used. Genetic samples from four consented couples in this study, together with scripts for assessing coverage and accuracy of embryo prediction and generating figures in the article, can be found at https://github.com/myome/ivf_retrospective_pub.
