# Supplementary material for: Whole-genome risk prediction of common diseases in human preimplantation embryos
Source: Nat Med. 2022 Mar 21;28(3):513–6. doi: 10.1038/s41591-022-01735-0 (PMC8938270; doi:10.1038/s41591-022-01735-0)
Supplement: Supplementary file 1 — Supplementary Tables 1 and 2 and Notes 1–4. [file 41591_2022_1735_MOESM1_ESM.pdf]

nature portfolio

https://doi.org/10.1038/s41591-022-01735-0

Supplementary information

Whole-genome risk prediction of common diseases in human preimplantation embryos

In the format provided by the authors and unedited

Supplementary Information

Supplemental Tables

Supplemental Table 1. Whole-genome sequencing coverage statistics
Supplemental Table 2. Polygenic model and phenotype definitions in the UK Biobank

Supplemental Notes

Supplemental Note 1. Parental Support method for embryo genotype estimation
Supplemental Note 2. WGS with synthetic long read sequencing
Supplemental Note 3. Within-family polygenic risk score effect size
Supplemental Note 4. Centering approach for individuals with Ashkenazi Jewish Ancestry

Supplemental Data

Supplemental Table 1. Whole-genome sequencing coverage statistics.

	**Mother**		**Father**		**Child**	
**Case ID**	**Mean Depth**	**% ≥20X**	**Mean Depth**	**% ≥20X**	**Mean Depth**	**% ≥20X**
1	38X	97%	42X	96%	38X	95%
2	38X	96%	43X	96X	43X	97%
3	43X	98%	41X	95%	41X	97%
4	34X	92%	32X	85%	29X	83%
5	36X	96%	31X	90%	40X	97%
					41X	95%
6	38X	96%	30X	84%	34X	88%
7	39X	96%	30X	84%	39X	95%
8	36X	94%	34X	95%	29X	89%
9	47X	98X	43X	96%	37X	97%
10[1]	86X	98%	111X	99%	N/A	N/A

[1]Tell-Seq library prep. Mean Depth, average depth of coverage across the genome; % ≥20X, percentage of genomic bases covered by at least 20 sequence reads

Supplemental Table 2. Polygenic model and phenotype definitions in the UKBiobank[22]. PGS catalog (pgscatalog.org) identifiers provided when available.

Disease	(ICD10), (ICD9) codes	Phenotype terms (UKB data field, description, coding)	Source of polygenic model (PGS catalog ID where available)
Atrial fibrillation	(I48), (4273)	None	PGS000035[23]
Breast cancer	(C50, D05), (174, 2330)	(20001, self reported cancer, 1002)	PGS000008[24]
Coronary artery disease	(I20, I21, I22), (410, 411)	(20002, self reported, 1075)	PGS000054[25]
Colorectal cancer	(C18), (153)	(20001, self reported cancer, 1020, 1022)	PGS000074[26]
Crohn's disease	(K50)	(20002, non cancer self reported, 1462)	Liu et al; Huang et al[27,28]
Lupus	(M32), (710)	(20002, non cancer self reported, 1381)	Chen et al[29]
Pancreatic cancer	(C25), (157)	(20001, cancer self reported, 1026)	PGS000083[26]
Prostate cancer	(C61), (185)	(20001, cancer self reported, 1044)	PGS000030[30]
Type 1 diabetes	(E10), (25001, 25011, 25021, 25091)	(20002, self reported, 1222), all conditioned on (2976, age of diabetes diagnosis, < 35)	Oram et al[31]
Type 2 diabetes	(E11), (25000, 25010, 25020, 25090, 2503, 2504, 2505, 2506, 2507)	(2443, diabetes diagnosed by doctor, 1), (6177, medications for blood pressure, diabetes, etc, 3), all conditioned on (2976, age of diabetes diagnosis, > 35)	PGS000020[32]
Ulcerative colitis	(K51)	(20002, non cancer self reported, 1463)	Liu et al; Huang et al[27,28]
Vitiligo	(L80)	(20002, non cancer self reported, 1661)	Roberts et al[33]

Supplemental Methods

Supplemental Note 1: Parental Support method for embryo genotype estimation

Approach to cleaning noisy genetic measurements from embryo biopsies: “Parental Support Approach”

	Page
Background	4
Simplified example	4
Hidden Markov Model and related parameters	8
Calculating Transmission Probabilities	10
Calculating Emission Probabilities (Emission Models)	10
Parameter estimates used in HMM	12
Parental Support Results	12
Genome Prediction in Embryo	14

Background

Whole genome reconstruction as discussed in the manuscript involves accurate determination of a subset of genotypes derived from microarray measurements from single or few cell biopsies from embryos preimplantation (Figure N1A; “PS Embryo Genotypes”; Table N1). “Parental Support”, also referred to as the phasing algorithm in this supplement, is a method of combining SNP array measurements from multiple embryos and the parents along with recombination frequencies from the HapMap database to enable accurate prediction of chromosome copy numbers, insertions and deletions, embryo genotypes, parent haplotypes as well as embryo parent haplotype origin hypotheses. We describe the process in US Patent 8515679_B2. A summary of the method with certain updates follows. Note that phasing and reconstruction is completed only for euploid chromosomes.

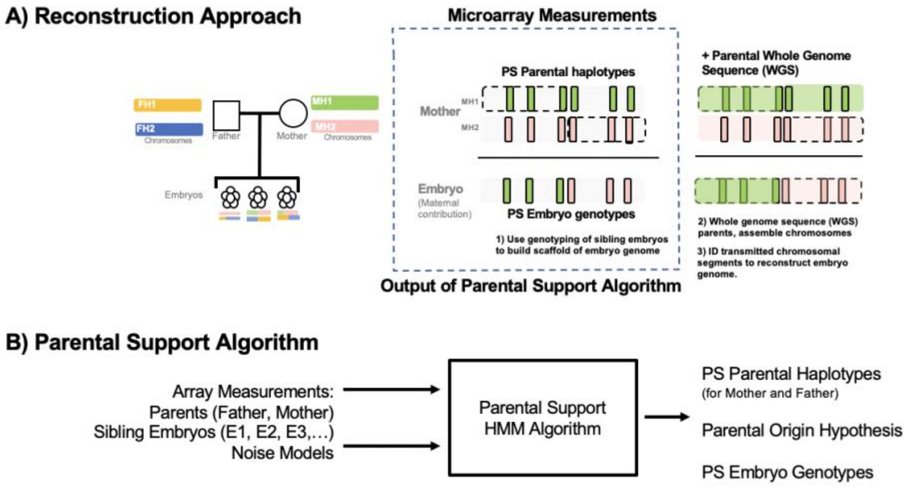

Figure N1A. Whole genome reconstruction involves two sources of data. 1) Whole genome sequencing of prospective parents and 2) SNP microarray genotyping of sibling embryos. Limited DNA in embryo biopsy requires amplification and results in inaccuracies in sequence. **Figure N1B.** Allele measurements at each SNP are color-coded based on the parental haplotype of origin in this example. The Parental Support algorithm uses an HMM that accounts for measurements on sibling genotypes as well as parental genotypes to improve accuracy across several hundred thousand positions. The output of this approach are PS Parental Haplotypes and PS Embryo Genotypes at Illumina CytoSNP12b array locations.

**Parental Support Microarray Inputs**	Parental Microarray Data	Mother (MD) and father (FD) microarray data, with genotypes marked as AA,AB,BB (A=Ref,B=Mut).
	Embryo Microarray Data	Up to k Embryo microarray measurements ($ED_1,...,ED_k$), stored as intensity values on the "A" channel and "B" channel.
	We denote the full set of data below as :$D=(D_1,...,D_n)=(MD_t,FD_t,ED_{tj})$, t=1,...,n, j=1,...,k.	
**Parental Support Outputs**	PS Parental Haplotypes	A best estimate of each phased parental genotype, denoted as (genotype at haplotype1,genotype at haplotype 2).<br><br>E.g. phased genotype AB signifies that A comes from the first haplotype and B from the second. For each position on the microarray, the possible phased parental haplotypes are {AA, AB, BA, BB}.
	Embryo Parental Origin Hypothesis	The particular haplotypes inherited from the specific parent. Denoted as H1 or H2, depending on which haplotype the embryo inherited from a parent.<br>E.g. MH1,FH2 means that the embryo has inherited its mother's haplotype 1 and father's haplotype 2.
	PS Embryo Genotype	A direct combination of mother, father haplotypes and parental origin hypothesis. Calculated only on euploid segments of the genome.<br>E.g. if parent haplotypes are (BA,AB) and parent origin hypotheses are (H1,H2), the embryo inherits a B from mother (H1 from BA) and a B from father (H2 from AB), so the PS embryo genotype is BB.

Table N1. Definitions of terms used

<table>
<tr><th colspan="2">Parent Data</th><th colspan="6">PS algorithm usage</th></tr>
<tr><th>parent context</th><th>context name</th><th>embryo genotype</th><th>allele queried</th><th>Discrete emission model</th><th>Continuous emission model</th><th>parent haplotypes</th><th>parent origin hypotheses</th></tr>
<tr><td>BB|BB</td><td>no signal A</td><td>BB</td><td>A</td><td>noise floor A</td><td>model given BB</td><td>(BB|BB)</td><td></td></tr>
<tr><td>AA|AA</td><td>no signal B</td><td>AA</td><td>B</td><td>noise floor B</td><td>model given AA</td><td>(AA|AA)</td><td></td></tr>
<tr><td>AA|BB, BB|AA</td><td>polar AB,BA</td><td>AB</td><td>A,B</td><td>allele dropout</td><td>model given AB</td><td>(AA|BB), (BB|AA)</td><td></td></tr>
<tr><td>(AB|BB)</td><td>signal MA</td><td>AB or BB</td><td>A</td><td></td><td></td><td>(AB or BA,BB)</td><td>mother origin</td></tr>
<tr><td>(BB|AB)</td><td>signal FA</td><td>AB or BB</td><td>A</td><td></td><td></td><td>(BB,AB or BA)</td><td>father origin</td></tr>
<tr><td>(AB,AA)</td><td>signal MB</td><td>AB or AA</td><td>B</td><td></td><td></td><td>(AB or BA,AA)</td><td>mother origin</td></tr>
<tr><td>(AA,AB)</td><td>signal FB</td><td>AB or AA</td><td>B</td><td></td><td></td><td>(AA,AB or BA)</td><td>father origin</td></tr>
<tr><td>(AB,AB)</td><td>mixed</td><td>AA,AB or BB</td><td>A,B</td><td></td><td></td><td>(AB or BA,AB or BA)</td><td>mother,father origin</td></tr>
</table>

Table N2. Parent context scenarios and PS algorithm usage

Simplified example

To conceptually illustrate how noisy genetic measurements from sibling embryos and parents can be leveraged to increase accuracy, we start with a simplified approach on one chromosome, assuming a) 4 embryos b) accurate parental genotypes c) no meiotic recombinations in the parents and d) noise in the embryo genotypes and only one chromosome inherited from each parent.

.

We introduce the concept of "Parent Context", which is the combination of mother and father genotypes at a particular site/SNP, denoted as (mother genotype|father genotype). Specific contexts will inform the PS algorithm differently. Table N2 presents the possible scenarios of SNP parent contexts and the way in which they inform the PS algorithm. Table N3 describes an example implementation.

	**GENOTYPE DATA**						**PS OUTPUT**						
	Parental Genotypes		Raw Embryo Genotypes (Noisy)				PS Parental Haplotypes		PS Embryo Genotypes				
Embryo Parent Origin Hypotheses									(1,1)	(2,2)	(2,1)	(1,1)	
SNP	mother	father	E1	E2	E3	E4	mother	father	e1	e2	e3	e4	Step
1	AA	AA	AA	AA	AA	AB	AA	AA	AA	AA	AA	AA	STEP1
2	AA	BB	AB	AB	BB	AB	AA	BB	AB	AB	AB	AB	STEP1
3	AB	BB	AB	BB	BB	AB	AB	BB	AB	BB	BB	AB	STEP2
4	AA	AB	AA	AB	AA	AA	AA	AB	AA	AB	AA	AA	STEP2
5	AB	AB	AB	AB	AA	AB	BA	AB	AB	AB	AA	AB	STEP4
6	AB	BB	AB	AA	AB	BB	BA	BB	BB	AB	AB	BB	STEP3
7	AB	AB	AA	AB	AA	AB	BA	AB	BA	AB	AA	BA	STEP4
8	AB	BB	AB	AB	AB	AB	AB/BA	BB	?B	?B	?B	?B	STEP3

Table N3. Sample Data and PS output. Red letters indicate scenarios where PS Embryo Genotypes differ from the raw embryo genotypes.

STEP 1 Set homozygous parents and unambiguous parent contexts on SNPs 1,2, so correcting mendelian errors in e3 on SNP 1 and e4 on SNP 2. By symmetry, we also set the phase (PS Parental Haplotype) of the first heterozygous mother (SNP 3) and father (SNP 4) to AB.

STEP 2 Compute most likely embryo hypotheses for all SNPs with currently phased parents -- SNPs 3,4 here. We present possible parent origin hypothesis scenarios, and choose the most likely scenario for each embryo, as seen in Table N4A.

Embryo Parent Origin Hypotheses		(SNP3,SNP 4) possible embryo outcome	# SNPs matching with each hypothesis			
MH	FH		E1	E2	E3	E4
1	1	(AB,AA)	2	0	1	2
1	2	(AB,AB)	1	1	0	1
2	1	(BB,AA)	1	1	2	1
2	2	(BB,AB)	0	2	1	0
**Best Embryo Parent Origin Hyp. (MH,FH) for each embryo**			**(1,1)**	**(2,2)**	**(2,1)**	**(1,1)**

Table N4A. Step 2 scenarios

STEP 3 Phase parents at sites where only one parent is ambiguous, using embryo genotypes and putative parent origin hypotheses for E1 through E4 (determined in Step2) resulting in the most likely estimate of PS Maternal Haplotypes for SNPs 6 and 8. The best match is found on SNP 6 and E1, E2 corrected, as seen in Table N4B.

Maternal Haplotype Alleles		Resulting Embryo genotype (given father=BB & hypotheses from Step 2)				# embryo matches for each SNP	
		E1	E2	E3	E3	SNP 6	SNP 8
Maternal haplotype scenarios	AB	AB	BB	BB	AB	1	2
	BA	BB	AB	AB	BB	2	2
BEST for SNP 6	BA	BB	AB	AB	BB		
BEST for SNP 8	AB/BA	?B	?B	?B	?B		

Table N4B. Step 3 scenarios

In this simplified case both maternal scenarios are equally likely on SNP 8. In the case of embryo data given by microarray measurements, we will get one scenario more likely than the other with confidence adjusted accordingly.

STEP 4 Phase parents at sites where both parents are ambiguous, using embryo genotypes and putative parent origin hypotheses for E1 through E4 (determined in Step2). This results in the most likely estimate of PS Parental Haplotypes for SNPs 5 and 7. PS finds the best matches for both SNPs and corrects E1 on SNP 7, as seen in Table N4C.

scenarios	MG	FG	E1	E2	E3	E3	#match SNP 5	#match SNP 7
	AB	AB	AA	BB	BA	AA	0	1
	AB	BA	AB	BA	BB	AB	3	2
	BA	AB	BA	AB	AA	BA	4	3
	BA	BA	BB	AA	AB	BB	0	0
best SNP 5	BA	AB	AB	AB	AA	AB		
best SNP 7	BA	AB	BA	AB	AA	BA		

Table N4C. Step 4 scenarios

Final Output PS Parental Haplotypes, PS Embryo genotypes and parent origin hypotheses are given in Table N3 (Column "PS Output").

Hidden Markov Model and related parameters

The full implementation of Parental Support supporting meiotic crossovers involves a Hidden Markov Model (HMM) with a forward-backward (FBA) algorithm implemented.

Background: A Hidden Markov Model (HMM) is a statistical Markov model in which the system being modeled is assumed to be a Markov process X_t, through "time" t, with unobservable, i.e. hidden states {x}. The approach assumes that there is another process Y_t, with observable states {y}, whose behavior through time depends on X (Figure N2A and N2B). The goal is to learn about X by observing Y. In an HMM we assume that for each time instance t, the conditional distribution of Y_t depends only on X_t, via probability $P(y|x)=P(Y_t=y|X_t=x)$. This probability is the emission probability.

The probability of the observable sequence $Y=(Y_1,...,Y_n)$ can be written by Bayes rule as $P(Y)=\sum_X P(Y|X)P(X)$.

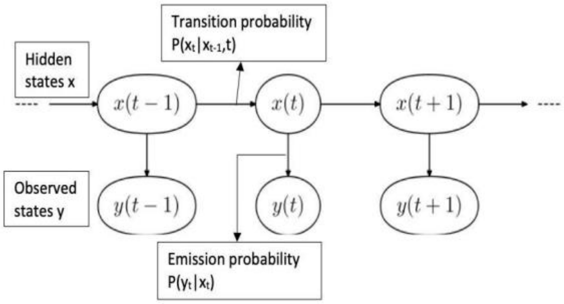

Figure N2A. Hidden Markov Model setup

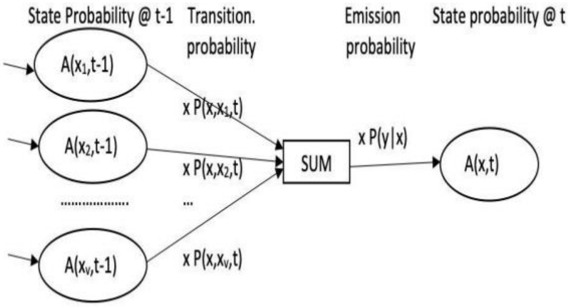

Figure N2B. HMM calculation

For an HMM as in Figure N1A, we are interested in the posterior probability $P(X_t=x|(y_1,..,y_n))$, i.e. a probability of an unobservable state x at time t, given observed states $(y_1,..,y_n)$. The forward algorithm calculates the joint probability of a hidden state x and $(y_1,..,y_t)$ $A(x,t)=P(X_t=x,y_1,..,y_t)$ as $A(x,t)=P(Y_t=y_t|X_t=x)*\sum_z P(x|z,t)*A(z,t-1)$ thus reducing the problem of order t to the problem of order t-1, as seen in Figure N1B. P(x|z,t) is referred to as a hidden state transition probability at time t. Posterior probability of any hidden state x at time n is then $P(x|(y_1,..,y_n))\sim A(x,n)$.

Specific Implementation for Embryo Measurements (Parental Support): This statistical model incorporates the fact that an embryo will inherit alleles from the same parent homolog on consecutive SNPs, unless a meiotic recombination (with probability estimated in the HapMap database) has occurred between the two SNPs. The joint distribution on genotype probabilities thus combines the array data, the individual embryo genotypes suggested by the array data, and the parent haplotyping that could produce those distributions of genotypes among various embryos. Consecutive SNPs represent "time" t, with additional definitions below as shown in Figure N3 and Table N5. The approach is applied to each full chromosome separately, at all sites on the array. The number of SNPs per chromosome ranges from 4.3K (chrom 21) to 23.7K (chrom 2). In an advance over Kumar et al. 2015 the approach is run across the entire chromosome instead of smaller regions of the genome. This modified approach allows for crossovers within, as well as between bins, as well as inference of problematic genome sections.

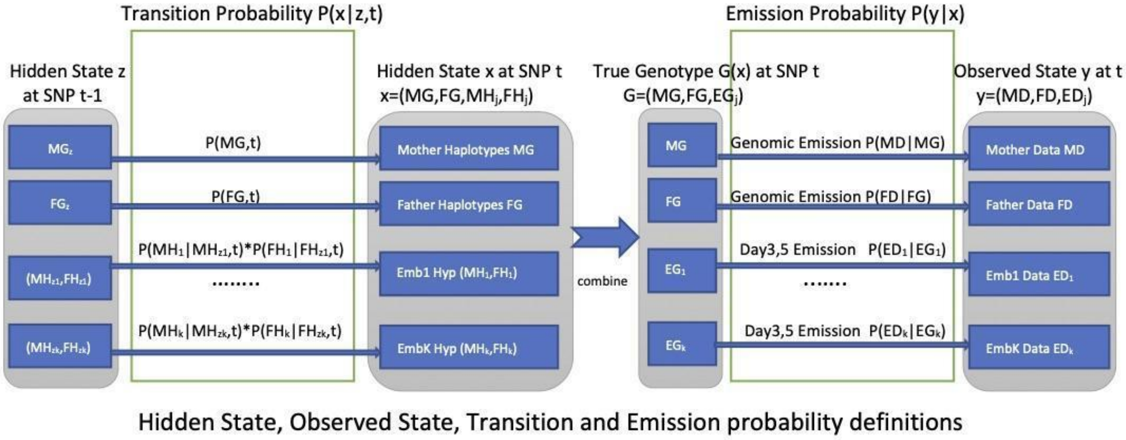

Figure N3. HMM framework ("Parental Support") used to calculate PS Embryo Genotypes and PS Parental Haplotypes from microarray measurements on sibling embryos and parents. Between-SNP Transition probabilities are calculated using genetic maps and databases of recombination frequency.

**Observed State**	y_t is the combination of parent and embryo microarray measurements at a particular SNP, $y_t=(MD_t,FD_t,ED_{tj})$ for j=1,...,k.
**Hidden State**	x is defined as a combination of true parent haplotypes and parent origin hypotheses $x=(MG,FG,MH_1,FH_1,...,MH_k,FH_k)$ where $MG,FG\in\{AA,AB,BA,BB\}$, the set of parent haplotype pairs and $MH_j,FH_j\in\{H1,H2\}$, the set of parental origin hypotheses.
**Transition probability**	The product of haplotype prior frequencies (assuming no parent SNP linkage) and origin hypotheses transition probabilities. Specifically, for states $x=(MG,FG,MH_j,FH_j,j=1...k)$ at SNP t and state $z=(MG_z,FG_z,MH_{zj},FH_{zj},j=1...k)$ at time t-1
**Emission probability**	P(y|x) is defined as a product of parent and embryo SNP data likelihood (emission probabilities), given the true state x<br>$P(y\|x) = P(MD\|MG) \times P(FD\|FG) \times \prod_{j=1,...,k} P(ED_j\|EG_j)$,<br>where EG_j is the jth embryo genotype, a direct combination of parent haplotypes MG,FG and jth parent origin hypotheses (MH_j,FH_j), as mentioned before. P(MD|MG), P(FD|FG) are parent data genomic emission models and P(ED|EG) is the embryo data emission model, further discussed below.

Output: PS Parental Haplotype	Parent haplotypes probability is the marginal of the joint probability P(x|y), for all states having specific parent haplotype, i.e. $P(MG|y)=\sum_{x\in MG}P(x|y)$. For each parent, we derive the best answer g* as the one maximizing this probability, i.e. $g^*=\text{argmax}_g P(g|y)$, with confidence P(g*).
**Output: Parental Origin Hyp.**	The state maximizing the marginal parent origin hypothesis probability $P(H|y)=\sum_{x\in H}P(x|y)$.

Table N5. Additional Definitions related to the Parental Support HMM.

Calculating Transmission Probabilities

In the simplified approach described above, we assumed that all parental haplotypes are inherited in the embryos without recombination. To model the meiotic recombinations between consecutive SNPs we compute the transmission probability from state z at SNP t-1 to state x at SNP t as

$$P(x \text{ at SNP } t|z \text{ at SNP } t\text{-}1)=P(MG,t)*P(FG,t)*\prod_{j=1,\ldots,k}P(MH_j|MH_{zj},t)xP(FH_j|FH_{zj},t), \text{ where:}$$

- P(MG,t) and P(FG,t) are parent haplotypes population priors at SNP t, derived from a large set of training data and allele frequency public databases.
- $P(MH|MH_z,t)$, $P(FH|FH_z,t)$ are the hypotheses transition probabilities, derived via crossover probabilities between SNPs t-1 and t, from the HapMap database, simulating a chance of meiotic crossover between SNPs. Specifically, $P(H1|H1,t)=P(H2|H2,t)=1-c_t$ (no crossover occurred) and $P(H1|H2,t)=P(H2|H1,t)=c_t$ (crossover occurred), where c_t=crossover probability between SNPs t-1 and t, derived via HapMap database.

Calculating Emission Probabilities (Emission Models)

Noise in the microarray measurements of parent or embryo are accounted for in the "emission model" of the HMM. Specifically, the emission probabilities are the per SNP product of per channel data likelihood given a true genotype G: P(Data|genotype=G)=P(Data on channel A|G)*P(Data on channel B|G). We use two different approaches to modeling channel data likelihood: a simplified discrete emission model and a more complex continuous emission model (Figure N4).

The Discrete Emission Model is defined as the channel independent matrix product:

$$F_{din,dout}(g,G)=P(\text{genotype}=g|\text{true genotype}=G, ADI, ADO)=P(\#A(g)|\#A(G))*P(\#B(g)|\#B(G))$$

parameterized using a drop in rate (ADI) and drop out rate (ADO), based on number of alleles A,B in true genotype G and measured genotype g, as shown in Table N6. Dropin (ADI) and dropout (ADO) rate parameters are fit on a case-by-case basis using microarray intensity data, as follows.

# alleles in G (true)	#alleles in g (measured) 0	1	2
0	1-ADI	ADI	ADI
1	ADO	1-ADO	1-ADOt
2	ADO^2	ADO	$1-ADO^2$

Table N6. Discrete emission model matrix $F_{ADI,ADO}(g,G)$

First we determine a channel "noise floor" as the 95th percentile of channel array measurements for SNPS where no channel signal should be present, such as SNPS with same homozygous parents, context (AA|AA) for channel B and (BB|BB) for channel A. We have effectively fixed the drop in rate at ADI=5%.
Second, we calculate the channel dropout rate (ADO) as the percent channel measurements less than "noise floor" out of all SNPS with guaranteed heterozygous genotype, such as "polar" SNPS with context (AA|BB) or (BB|AA). This process is demonstrated for one of 'case 10' embryos in Figure N3 ("Discrete Response").

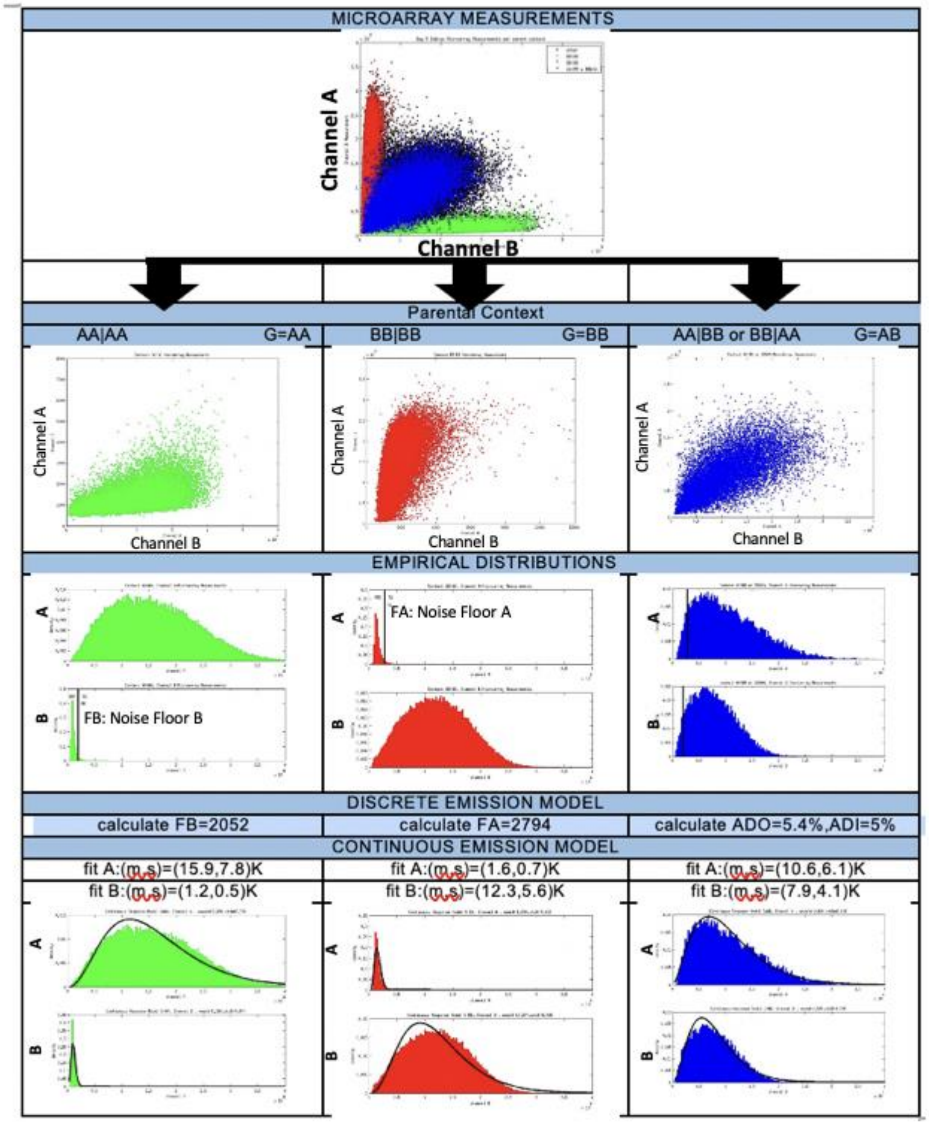

Figure N4. Calculating Emission Probability parameters, continuous and discrete approach for one Day 5 embryo (Case 10). Microarray measurements are colored by parental context. Noise floor FA and FB are used in the discrete emission model.

Continuous Emission Model: In this case, data measurements are modeled using a two-dimensional likelihood P(Data|G)=P(Channel A Measurement|G)*P(Channel B Measurement|G), where each channel likelihood is parameterized via known, continuous distribution for given G. Distribution parameters are

fitted in each couple using embryo microarray measurements for parental context resulting in G. The process is demonstrated for one of 'case 10' embryos in **Figure N4.**

Parameter estimates used in HMM

Genomic Data Emission Model For mother and father microarray data, we adopt discrete emission model with fixed parameter values, such as genomic data ADI rate=0.1% and genomic data ADO=0.15%, determined from a large training set of genomic samples.

Embryo Data Emission Model For embryo Day 3 or Day 5 microarray data, we use either discrete model (simple case), or continuous model (more accurate), with parameters determined on per embryo basis. Mean dropout rates, for Day 3 and 5 embryos in our study, are given in Table N7. A boxplot of estimated allele dropout rates for 90 Day 5 and 20 Day 3 embryos are given in Figure N5. Of note, only euploid chromosomes are displayed. If an embryo contains 23 chromosomes and one is aneuploid, only 22 chromosomes would be included. For simplicity, the Y chromosome was not considered in this analysis.

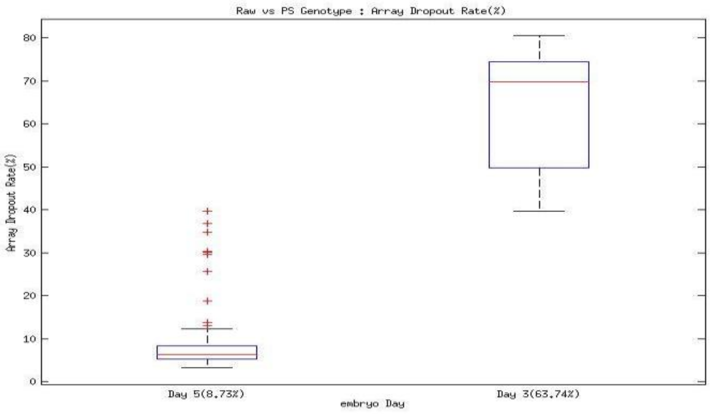

Figure N5. Estimated allele dropout rates across Day 5 and Day 3 embryos. Boxplots show median (red line), interquartile range(box), range not considered outliers(whiskers), and outliers(red crosses).
Day 5 rates, from n=90 samples, have a mean=8.7%, median= 6.2%, st. dev=7.5%, interquartile range of [5.2-8.3]%, range of [3.2%-39.6]%.
Day 3 rates, from n=20 samples, have a mean=63.7%, median= 69.8%, st. dev=13.7%, interquartile range of [49.8-74.5]%, range of [39.7-80.6]%.

Parental Support Results

We next examined the percent of total array sites correctly predicted both without parental support (raw genotype) or with parental support (PS embryo genotype). This analysis considered only euploid chromosomes. An average of 97% of the array sites are called with Day 5 embryo biopsies using parental support (PS embryo genotypes before cleaning with population data) vs. 68 % of array sites (n=150,000) without. Similarly, in Day 3 embryos 79% of array sites are called vs. 51% without. The accuracy of these calls is 99.5% for Day 5 embryos and 97% for Day 3 embryos, as opposed to 97.4% (Day 5) and 53.6% (Day 3) for microarray sites, an increase of 2% and 44% respectively. Mean dropout rates, coverage and correct call rates for raw and PS genotypes, along with dropout rates are given for all Day 5 (90 embryos) and Day 3 (20 embryos) in Table N7. Boxplots of rates for genotype kind and Day 5 vs Day 3 are given in Figure N6A and B. Parental

Support consistently increased the accuracy and coverage of predictions. Day 3 embryos had a higher drop out rate, lower coverage and lower accuracy compared with Day 5 embryos. PS Embryo genotypes are further processed in our approach of whole genome reconstruction (**Figure N1A**) and below, to increase the number of sites predicted in both Day 3 and Day 5 embryos (see **Extended Data Fig. 1).**

	**Total #**	**Allele Dropout Rate (%)**	**Coverage(%)**		**Correct Call Rate(%)**	
			**REG**	**PS**	**REG**	**PS**
**Day 5**	90	8.7	68.3	96.7	97.4	99.5
**Day 3**	20	64.2	51.2	79.1	53.6	97

Table N7. Rates for regular (raw) array embryo genotypes and PS embryo genotypes for Day 5 and 3 embryos

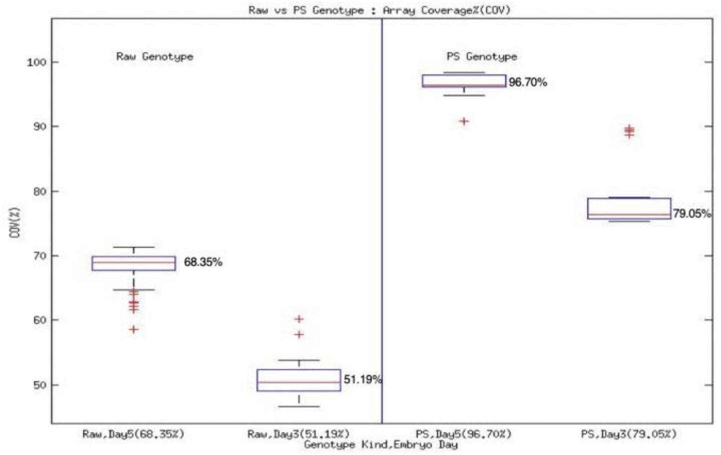

B)

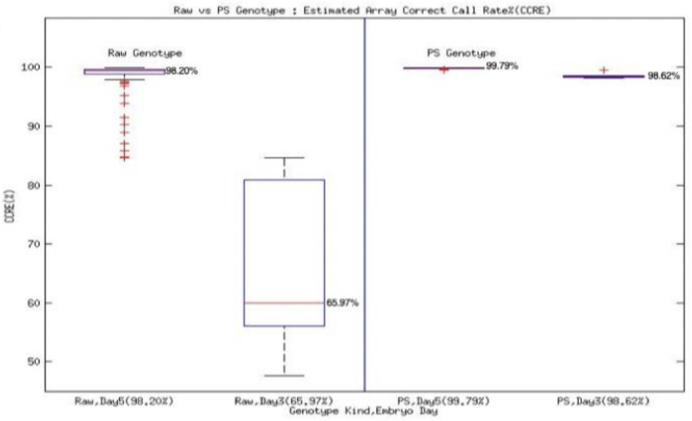

Figure N6A. Estimated allele dropout rates across Day 5 and Day 3 embryos. Boxplots show median (red line), interquartile range(box), range not considered outliers(whiskers), and outliers(red crosses).

Day 5 rates, from n=90 samples, have a mean=8.7%, median=6.2%, st. dev= 7.5%, interquartile range of [5.2-8.3]%, range of [3.2%-39.6]%.

Day 3 rates, from n=20 samples, have a mean=63.7%, median=69.8%, st. dev=13.7%, interquartile range of [49.8-74.5]%, range of [39.7-80.6]%.

N6B. Estimated allele dropout rates across Day 5 and Day 3 embryos. Boxplots show median (red line), interquartile range(box), range not considered outliers(whiskers), and outliers(red crosses).

Day 5 rates, from n=90 samples, have a mean=8.7%, median=6.2%, st. dev=7.5%, interquartile range of [5.2-8.3]%, range of [3.2%-39.6]%.

Day 3 rates, from n=20 samples, have a mean=63.7%, median=69.8%, st. dev=13.7%, interquartile range of [49.8-74.5]%, range of [39.7-80.6]%.

Genome Prediction in Embryo

We used the PS Parental Haplotypes and PS Embryo Genotypes in combination with whole genome sequencing of each parent as described in the methods section to enable whole genome prediction of each embryo. See example predictions of transmitted segments in **Figure N7 b**elow. Briefly, we phased WGS-identified variants (~6 million) into "phased parental genomes" using PS Parental Haplotypes and a population reference panel. We computed which portion of the parent's chromosomes was transmitted to the embryo by comparing the PS haplotype with the PS embryo genotypes and repeated the process across all maternal and paternal chromosomes (see **Extended Data Fig. 7-8**). Results of the reconstruction approach can be found in **Extended Data Fig. 1**.

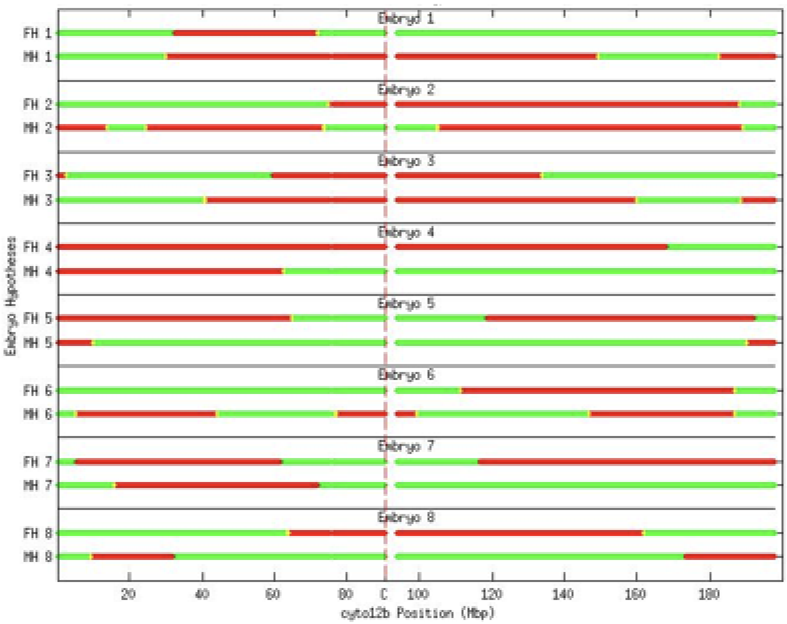

Figure N7: Here is a plot of transmitted haplotypes on chromosome 3 across 8 sibling embryos derived from family 5. Transmitted haplotypes were output from parental support and form the basis of the PS Embryo Genotypes at microarray sites. Green and red lines denote parental haplotype 1 and 2 respectively

for mother (MH) and father (FH) haplotypes in each embryo. (Regions of some uncertainty are colored yellow).

Supplemental Note 2. WGS with synthetic long read sequencing

Linked read sequencing data was generated for Case IDs 5, 8, 9, and 10 using the TELL-Seq library preparation method. After read alignment and variant calling using the same process described above with the addition of maintaining molecular barcode information for each read, we inferred the molecular phase using HapCut2[34] with default parameters except maxFragments=1000000 and d=40000. We annotated positions with their global allele frequency using the gnomad database[35].

Supplemental Note 3: Within-family polygenic risk score effect size

To examine within family effects of PRS, we applied a random intercept mixed-effects model, similar to Selzam et al 2019, on a total of 9,000 sibling pairs within the UK Biobank, including two fixed effects to separate within and between family effects:

$$logit\, p_{ij} = \alpha + \beta_W(PRS_{ij} - \overline{PRS}_j) + \beta_F\overline{PRS}_j + \gamma_j$$

Where β_W is the within family slope, PRS_{ij} is the PRS in individual i and family j, $PRSj$ is the average PRS for family j, β_F is the slope between families, and γ is a random intercept term. Our analysis did not find a significant difference in breast cancer PRS effect sizes for siblings vs. unrelated individuals. Although the UK Biobank does not have enough siblings to repeat this analysis across all diseases, we anticipate similar findings for most diseases.

Supplemental Note 4: Centering approach for individuals with Ashkenazi Jewish Ancestry

For individuals with AJ ancestry, we found the above method that relies on principal component analysis of 1000 Genomes individuals did not sufficiently correct for ancestry, likely due to low representation of AJ individuals in the 1000 Genomes project. Thus, for individuals with AJ ancestry, we center and standardize using a population of AJ identified in the UK Biobank, following the approach detailed in Prive et al 2021[36]. Specifically, we project data from UK Biobank participants onto principal components calculated using the Khazar dataset from Behar et al[37]. We then identify the geometric median of the AJ reference individuals and calculate the distance to this center for all UKB individuals. We then chose a threshold such that all AJ individuals in the reference set are included and all other populations excluded. 470 UKB individuals were assigned to this group as AJ. We use the mean and standard deviation of the scores in this group of individuals to center and standardize the raw PRS for AJ individuals in the study.

References

22. Sudlow, C. *et al.* UK biobank: an open access resource for identifying the causes of a wide range of complex diseases of middle and old age. *PLoS Med.* **12**, e1001779 (2015).

23. Weng, L.-C. *et al.* Genetic Predisposition, Clinical Risk Factor Burden, and Lifetime Risk of Atrial Fibrillation. *Circulation* **137**, 1027–1038 (2018).

24. Mavaddat, N. *et al.* Polygenic Risk Scores for Prediction of Breast Cancer and Breast Cancer Subtypes. *Am. J. Hum. Genet.* **104**, 21–34 (2019).

25. Morieri, M. L. *et al.* Genetic Tools for Coronary Risk Assessment in Type 2 Diabetes: A Cohort Study From the ACCORD Clinical Trial. *Diabetes Care* **41**, 2404–2413 (2018).

26. Graff, R. E. *et al.* Cross-Cancer Evaluation of Polygenic Risk Scores for 17 Cancer Types in Two Large Cohorts. doi:10.1101/2020.01.18.911578.

27. Liu, J. Z. *et al.* Association analyses identify 38 susceptibility loci for inflammatory bowel disease and highlight shared genetic risk across populations. *Nat. Genet.* **47**, 979–986 (2015).

28. Huang, C. *et al.* Genetic Risk for Inflammatory Bowel Disease Is a Determinant of Crohn's Disease Development in Chronic Granulomatous Disease. *Inflamm. Bowel Dis.* **22**, 2794–2801 (2016).

29. Chen, L. *et al.* Genome-wide assessment of genetic risk for systemic lupus erythematosus and disease severity. *Hum. Mol. Genet.* **29**, 1745–1756 (2020).

30. Schumacher, F. R. *et al.* Association analyses of more than 140,000 men identify 63 new prostate cancer susceptibility loci. *Nat. Genet.* **50**, 928–936 (2018).

31. Oram, R. A. *et al.* A Type 1 Diabetes Genetic Risk Score Can Aid Discrimination Between Type 1 and Type 2 Diabetes in Young Adults. *Diabetes Care* **39**, 337–344 (2016).

32. Läll, K., Mägi, R., Morris, A., Metspalu, A. & Fischer, K. Personalized risk prediction for type 2 diabetes: the potential of genetic risk scores. *Genet. Med.* **19**, 322–329 (2017).

33. Roberts, G. H. L., Paul, S., Yorgov, D., Santorico, S. A. & Spritz, R. A. Family Clustering of Autoimmune Vitiligo Results Principally from Polygenic Inheritance of Common Risk Alleles. *Am. J. Hum. Genet.* **105**, 364–372 (2019).

34. Edge, P., Bafna, V. & Bansal, V. HapCUT2: robust and accurate haplotype assembly for diverse sequencing technologies. *Genome Research* vol. 27 801–812 (2017).

35. Karczewski, K. J. *et al.* The mutational constraint spectrum quantified from variation in 141,456 humans. *Nature* **581**, 434–443 (2020).

36. Privé, F. *et al.* High-resolution portability of 245 polygenic scores when derived and applied in the same cohort. doi:10.1101/2021.02.05.21251061.

37. Behar, D. M. *et al.* No evidence from genome-wide data of a Khazar origin for the Ashkenazi Jews. *Hum. Biol.* **85**, 859–900 (2013).
