# Supplementary material for: Whole-genome risk prediction of common diseases in human preimplantation embryos
Source: Nat Med. 2022 Mar 21;28(3):513–6. doi: 10.1038/s41591-022-01735-0 (PMC8938270; doi:10.1038/s41591-022-01735-0)
Supplement: Supplementary file 2 — Reporting Summary [file 41591_2022_1735_MOESM2_ESM.pdf]

nature research

Corresponding author(s): Akash Kumar
Last updated by author(s): Sep 21, 2021

Reporting Summary

Nature Research wishes to improve the reproducibility of the work that we publish. This form provides structure for consistency and transparency in reporting. For further information on Nature Research policies, see our Editorial Policies and the Editorial Policy Checklist.

Statistics

For all statistical analyses, confirm that the following items are present in the figure legend, table legend, main text, or Methods section.

n/a	Confirmed	
☐	☒	The exact sample size (*n*) for each experimental group/condition, given as a discrete number and unit of measurement
☐	☒	A statement on whether measurements were taken from distinct samples or whether the same sample was measured repeatedly
☒	☐	The statistical test(s) used AND whether they are one- or two-sided<br>*Only common tests should be described solely by name; describe more complex techniques in the Methods section.*
☐	☒	A description of all covariates tested
☒	☐	A description of any assumptions or corrections, such as tests of normality and adjustment for multiple comparisons
☐	☒	A full description of the statistical parameters including central tendency (e.g. means) or other basic estimates (e.g. regression coefficient) AND variation (e.g. standard deviation) or associated estimates of uncertainty (e.g. confidence intervals)
☒	☐	For null hypothesis testing, the test statistic (e.g. *F*, *t*, *r*) with confidence intervals, effect sizes, degrees of freedom and *P* value noted<br>*Give P values as exact values whenever suitable.*
☒	☐	For Bayesian analysis, information on the choice of priors and Markov chain Monte Carlo settings
☒	☐	For hierarchical and complex designs, identification of the appropriate level for tests and full reporting of outcomes
☒	☐	Estimates of effect sizes (e.g. Cohen's *d*, Pearson's *r*), indicating how they were calculated

Our web collection on statistics for biologists contains articles on many of the points above.

Software and code

Policy information about availability of computer code

Data collection	No code was used to collect the data analyzed in this study.
Data analysis	For whole-genome sequence processing, Sentieon software (including bwa 0.7.17) was used to align sequence reads to genome reference and perform variant calling https://www.sentieon.com/. bcftools v.1.11-34 was used for variant filtering. To calculate polygenic risk scores, plink v1.9 and v2.0 https://zzz.bwh.harvard.edu/plink/ was used to calculate polygenic risk scores. Genetic samples from four consented couples in this study, together with scripts for assessing coverage and accuracy of embryo prediction and for generating figures in the manuscript, can be found at https://github.com/myome/ivf_retrospective_pub.

For manuscripts utilizing custom algorithms or software that are central to the research but not yet described in published literature, software must be made available to editors and reviewers. We strongly encourage code deposition in a community repository (e.g. GitHub). See the Nature Research guidelines for submitting code & software for further information.

Data

Policy information about availability of data

All manuscripts must include a data availability statement. This statement should provide the following information, where applicable:
- Accession codes, unique identifiers, or web links for publicly available datasets
- A list of figures that have associated raw data
- A description of any restrictions on data availability

Access to primary sequence data from a subset of participants (prospective trial) is controlled and cannot be made publicly available. Sequence data from twelve individuals corresponding to four consented couples can be found here: EGAS00001005619 and EGAS00001001020. Inquiries on how to access the UK10K imputation reference panel data can be made through the Wellcome Trust Sanger Institute (datasharing@sanger.ac.uk)Information on obtaining approval for access to UK Biobank data is available at www.ukbiobank.ac.uk/researchers. Gnomad data was downloaded from s3://gnomad-public-us-east-1/release/.

Field-specific reporting

Please select the one below that is the best fit for your research. If you are not sure, read the appropriate sections before making your selection.

☒ Life sciences ☐ Behavioural & social sciences ☐ Ecological, evolutionary & environmental sciences

For a reference copy of the document with all sections, see nature.com/documents/nr-reporting-summary-flat.pdf

Life sciences study design

All studies must disclose on these points even when the disclosure is negative.

Sample size	No sample-size calculations were performed for this study. Final sample size was determined by DNA availability and quality.
Data exclusions	Data for individuals with low quality DNA or sequencing data that did not meet internal QC thresholds were excluded.
Replication	Replication was not performed in this study. However, 3-fold cross-validation was used to avoid over-fitting of logistic regression model parameters for polygenic risk score validation.
Randomization	Randomization is not relevant because we are not testing outcomes or including covariates. We are assessing the accuracy of a genotype prediction method.
Blinding	Blinding was not relevant to this study because there is no grouping. All samples are analyzed uniformly for prediction accuracy.

Reporting for specific materials, systems and methods

We require information from authors about some types of materials, experimental systems and methods used in many studies. Here, indicate whether each material, system or method listed is relevant to your study. If you are not sure if a list item applies to your research, read the appropriate section before selecting a response.

Materials & experimental systems

n/a	Involved in the study	
☒	☐	Antibodies
☒	☐	Eukaryotic cell lines
☒	☐	Palaeontology and archaeology
☒	☐	Animals and other organisms
☐	☒	Human research participants
☒	☐	Clinical data
☒	☐	Dual use research of concern

Methods

n/a	Involved in the study	
☒	☐	ChIP-seq
☒	☐	Flow cytometry
☒	☐	MRI-based neuroimaging

Human research participants

Policy information about studies involving human research participants

Population characteristics	Past users of Natera/Gene Security Network Spectrum pre-implantation genetic testing who had either completed or were in the midst of undergoing in vitro fertilization.
Recruitment	Individuals were recruited for research at IVF centers before (retrospective) or after (prospective) they received PGT results through Natera (formerly Gene Security Network).
Ethics oversight	E&I West Coast Board Institutional Review Board #10176 (Natera) and WCG IRB protocol #20180294 and 20202676

Note that full information on the approval of the study protocol must also be provided in the manuscript.
